# Ocular Hypertension Results in Hypoxia within Glia and Neurons throughout the Visual Projection

**DOI:** 10.3390/antiox11050888

**Published:** 2022-04-29

**Authors:** Assraa Hassan Jassim, Nana Yaa Nsiah, Denise M. Inman

**Affiliations:** 1 Department of Pharmaceutical Sciences, Northeast Ohio Medical University, Rootstown, OH 44272, USA; assraa.jassim@utrgv.edu; 2 Department of Pharmaceutical Sciences, North Texas Eye Research Institute, University of North Texas Health Science Center, Fort Worth, TX 76107, USA; nanayaansiah@my.unthsc.edu

**Keywords:** Müller glia, microglia, astrocytes, retinal ganglion cells, glaucoma, hypoxia

## Abstract

The magnitude and duration of hypoxia after ocular hypertension (OHT) has been a matter of debate due to the lack of tools to accurately report hypoxia. In this study, we established a topography of hypoxia in the visual pathway by inducing OHT in mice that express a fusion protein comprised of the oxygen-dependent degradation (ODD) domain of HIF-1α and a tamoxifen-inducible Cre recombinase (CreERT2) driven by a ubiquitous CAG promoter. After tamoxifen administration, tdTomato expression would be driven in cells that contain stabilized HIF-1α. Intraocular pressure (IOP) and visual evoked potential (VEP) were measured after OHT at 3, 14, and 28 days (d) to evaluate hypoxia induction. Immunolabeling of hypoxic cell types in the retina and optic nerve (ON) was performed, as well as retinal ganglion cell (RGC) and axon number quantification at each time point (6 h, 3 d, 14 d, 28 d). IOP elevation and VEP decrease were detected 3 d after OHT, which preceded RGC soma and axon loss at 14 and 28 d after OHT. Hypoxia was detected primarily in Müller glia in the retina, and microglia and astrocytes in the ON and optic nerve head (ONH). Hypoxia-induced factor (HIF-α) regulates the expression of glucose transporters 1 and 3 (GLUT1, 3) to support neuronal metabolic demand. Significant increases in GLUT1 and 3 proteins were observed in the retina and ON after OHT. Interestingly, neurons and endothelial cells within the superior colliculus in the brain also experienced hypoxia after OHT as determined by tdTomato expression. The highest intensity labeling for hypoxia was detected in the ONH. Initiation of OHT resulted in significant hypoxia that did not immediately resolve, with low-level hypoxia apparent out to 14 and 28 d, suggesting that continued hypoxia contributes to glaucoma progression. Restricted hypoxia in retinal neurons after OHT suggests a hypoxia management role for glia.

## 1. Introduction

Glaucoma is a neurodegenerative disease that impacts the function of RGCs in transmitting visual information from the eye to the brain. Consequently, glaucoma is the second leading cause of blindness for millions of people around the world [1]. Accumulating research effort and recent findings in models of glaucoma have shown that low oxygen availability (hypoxia) and the subsequent reoxygenation exposes cells to severe stress and protein damage in the retina and ONH [2,3,4,5,6,7].

During hypoxia, cells initiate a variety of defense responses mediated by the hypoxia-inducible factors (HIFs). HIFs are heterodimeric transcription factors consisting of α and β subunits. HIF-β is a stable, oxygen-insensitive subunit and HIF-α has three oxygen-sensitive subunits (HIF-1α, HIF-2α, or HIF-3α) that together bind to the hypoxia response element (HRE) in the nucleus to promote the transcription of hypoxia-responsive genes [8]. HIF-3α has not been studied extensively, and it is currently unknown whether HIF-1α or HIF-2α has a greater impact on the visual system in glaucoma. In addition, it is still not known whether all cells express HIF-α subunits or if there is cellular specificity [9,10]. HIF-α enables metabolic adaptation to hypoxia through increased glycolysis via the upregulation of glucose transporters (GLUTs); GLUT1 and GLUT3 [11,12].

Significant hypoxia in the ONH after laser photocoagulation of the trabecular meshwork was shown within 1 and 3 days, though the IOP levels were higher than generally observed in vivo [3]. In a rat model of OHT in which IOP was raised to 40–43 mmHg for 5 and 10 days, no hypoxia was detected in microglia and neurons in the retina in spite of the magnitude of IOP elevation [13], suggesting that timing may be critical for hypoxia detection. Alternatively, the sensitivity of methods used to detect hypoxia could be a factor. We recently showed that 4 weeks of IOP elevation drove hypoxia induction across retinal layers and cell types (Müller glia, astrocytes, and microglia) and retinal ganglion cells (RGCs) after OHT [7]. Indeed, after 4 weeks of OHT, we showed a significant increase in *Hif-1*α and *Hif-2*α transcripts and HIF-1α protein in the retina, concurrent with the degeneration of RGCs and axons, impairment of anterograde axonal transport, and accumulation of reactive oxygen species (ROS) [7]. However, we detected no hypoxia at 2 weeks of OHT despite significant loss of RGCs, axons, and anterograde transport [7].

To detect hypoxia in that study, we used pimonidazole, a compound that forms covalent adducts in cells that have a partial pressure of oxygen less than 10 mm Hg. In the ON, we showed a significant increase in *Hif-1*α transcripts but no change in *Hif-2*α and no pimonidazole-positive cells after 2 or 4 weeks of OHT [7]. The observation of hypoxia after exposure to non-physiological levels of IOP, as well as evidence for hypoxia at times when the IOP is high but nevertheless stable (2 and 4 weeks after OHT), suggest there are unanswered questions regarding the elements responsible for low oxygen conditions in a system that requires oxygen for optimal function. There is a need for more sophisticated tools and a more extensive timeline of hypoxia detection.

Through a Cre–loxP-based fate-mapping strategy to genetically and irreversibly label hypoxic cells in which HIF-1α was activated (via stabilization of the oxygen-dependent degradation domain (ODD)), we investigated how early after IOP elevation hypoxia occurs, where it occurs, and how long it lasts. The timeline utilized was carefully chosen based on our findings and others in the literature [3,7,14]. This hypoxia fate-mapping strategy, combined with immunofluorescence labeling of specific cell types, allowed us to unambiguously identify cells exposed to hypoxia, as well as when they become exposed. This mapping of hypoxic cells is the first-time hypoxia after OHT is addressed so directly.

## 2. Materials and Methods

### 2.1. Animals

Both sexes of 2-month-old CAG-creERT2-ODD transgenic mice were used. These transgenic mice express a fusion protein comprised of the ODD domain of Hif-1α and a tamoxifen-inducible Cre-ERT2 driven by a ubiquitous CAG promoter (a hybrid promoter containing the CMV immediate-early enhancer and a modified chicken β-actin promoter) in every cell where Hif-1α is stabilized. Hif-1α stabilization occurs when the ODD fails to be degraded; degradation occurs in normoxic conditions. Therefore, a tdTomato (tdT) reporter gene marks every cell exposed to hypoxia (low oxygen). Since stabilization is at the level of HIF-1α protein, not a result of adduct formation, it is considered to be more accurate than pimonidazole [14]. CAG-creERT2-ODD transgenic mice were crossed with the Rosa26 floxed-stop tdT (R26R/tdT) reporter mice so that tamoxifen administration drove tdT expression in cells that contain stabilized Hif-1α. These mice will be referred to as CAG-ODD mice throughout the text. Mice were obtained from Dr. Liya Yin, then bred and housed in a specific pathogen-free barrier facility on a 12-h light-dark cycle at the Northeast Ohio Medical University (Rootstown, OH, USA). Mice had access to standard rodent chow ad libitum. All procedures were approved by the Institutional Animal Care and Use Committee (2019-0020, approved through 18 October 2022) and performed in accordance with the ARVO Statement for the Use of Animals in Ophthalmic and Vision Research.

### 2.2. Genotyping

Ear or tail snips were collected and sent to Transnetyx for genotyping. The Cre-ERT2-ODD forward primer (TTAATCCATATTGGCAGAACGAAAACG), Cre-ERT2-ODD reverse primer (CAGGCTAAGTGCCTTCTCTACA), and tdT primers were used to detect the Cre-ERT2-ODD construct and/or the tdT. Control mice in this study were littermates that were negative for the Cre-ERT2-ODD construct.

### 2.3. Tamoxifen Injection

Tamoxifen (300 µL of a 10 mg/mL solution in sunflower oil) was administered interperitoneally once/day for five days to initiate transcription of cre-recombinase in 2-month-old CAG-ODD mice.

### 2.4. Intraocular Pressure (IOP) Measurements

Ten IOP measurements per eye were taken in lightly anesthetized mice (2.5% isoflurane) using a TonoLab rebound tonometer (iCare Finland) prior to, and then weekly after, induction of OHT. For time points shorter than 1 week, IOP was measured prior to sacrifice. IOP integral (mmHg-days exposure over baseline) was also calculated [7].

### 2.5. Ocular Hypertension (OHT) Model

Magnetic microbeads (1.5 µL, 8 μm diameter; COMPEL COOH-Modified, UMC4001; Bangs Laboratories) were injected into the anterior chamber of both eyes via glass pulled micropipette attached to a microinjection system, and a neodymium magnet was used to guide the microbeads into the trabecular meshwork to block the outflow of aqueous humor and increase IOP. We used the magnetic bead model successfully to elevate IOP up to four weeks. To eliminate the confounding factor of contralateral eye effects on glial activation [15], separate animals served as control.

### 2.6. Determination of Visual Function

The Diagnosis visual testing system (Celeris) was used to measure visual evoked potential (VEP). For both, mice were dark adapted for ≥1 h, then anesthetized with Ketamine (100 mg/kg)-Xylazine (10 mg/kg) and placed prone on a heated stage. Electrodes were placed in the mouse’s cheek (reference), scalp at the base of the skull, and the base of the tail (ground). Bright luminance stimulators, with a light intensity of 1 cd*s/m^2^, were placed on each eye and the output of 600 sweeps was processed. VEP output was compared across groups by evaluating N1 (negative peak) and P2 (positive peak) amplitudes, and response amplitude (the amplitude from N1 trough to P2 peak amplitude). Terminal VEP was measured before sacrifice.

### 2.7. Hypoxia Detection by Pimonidazole

As previously described [7], 60 mg/kg pimonidazole hydrochloride was administered by intraperitoneal (IP) injection 90 min before animals were sacrificed to detect hypoxia. The subsequent immunofluorescence staining of tissue sections with an anti-pimonidazole antibody reveals the presence of hypoxic cells. Briefly, 4% paraformaldehyde-fixed retinas and ONs were cryosectioned, then incubated for 1 h in blocking agent (1% Triton X-100, 0.5% bovine serum albumin (BSA), 0.9% NaCl, and 5% donkey serum in 1% phosphate buffered saline (PBS); PBS-T-BSA). Slides were incubated with anti-pimonidazole antibody (FITC-conjugated mouse anti-pimonidazole, 1:200, Hypoxyprobe Green kit; Hypoxyprobe, Burlington, MA, USA) in blocking solution overnight. The following day, retinal and ON sections were rinsed in PBS and cover-slipped for imaging using a Leica DMi8 confocal microscope. Six slides (four sections/slide) were imaged. We added primary antibody against pimonidazole conjugated to FITC (1:200) to ON and retinal sections from mice that received no pimonidazole IP injection in order to affirm pimonidazole labeling specificity.

### 2.8. Perfusion and Tissue Preparation

Mice were killed 6 h, 3 d, 14 d, and 28 d after OHT with an overdose of sodium pentobarbital (Beuthanasia-D, 390 mg/kg, IP; Merck Animal Health, Baton Rouge, LA, USA), then perfused transcardially with 0.1 M PBS, then with 4% PFA. Retina, ON, and brain were harvested for histology and immunofluorescence.

### 2.9. Cryosectioning

Using a Leica cryostat, fixed and cryoprotected retinas and ONs were sectioned either sagittally (retinas) or longitudinally (ONs) at 10–15 µm.

### 2.10. Microtome Sectioning

Brains were coronally sectioned at 50 µm using a Leica freezing microtome (Wetzlar, Germany), then 10 representative sections were collected of the superior colliculus (SC), the primary and most distal site of the RGC projection in rodents; the hypothalamic suprachiasmatic nuclei (SCN), site of the master circadian pacemaker; and the lateral geniculate nucleus (LGN) of the thalamus, which receives inputs from both eyes and relays these messages to the primary visual cortex via the optic radiation. Sections were mounted on slides, imaged using a Zeiss AxioZoom V16 (AxioCam MRm Rev.3; Zeiss, Jena, Germany) and analyzed using ImageJ to calculate tdT mean intensity [7].

### 2.11. Immunofluorescence (IF)

Cryosectioned retina and ON were immunolabeled using the following antibodies: RNA binding protein multiple splicing (RBPMS, an RGC marker, 1:300, GeneTex, Irvine, CA, USA, GTX118619), Protein Kinase C alpha (PKCα, a bipolar cell marker, 1:250, Santa Cruz Biotechnology, Dallas, TX, USA sc-8393), glial fibrillary acidic protein (GFAP, an astrocyte and Müller glia marker, 1:500, Abcam, Cambridge, MA, USA, ab53554), ionized calcium binding adaptor molecule 1 (Iba-1, a microglia marker, 1:300, Wako, Richmond, VA, USA, 019-10741), choline acetyltransferase (ChAT, an amacrine cell marker, 1:200, Millipore, Burlington, MA, USA, AB144p), CD-31 (an endothelial cell marker, 1:50, Novus Biologicals, Littleton, CO, USA, NB100-2284) and vimentin (Vim, a Müller glia marker, 1:250, Novus Biologicals, Littleton, CO, USA, NB300-223). To determine the above dilutions, optimization with different serial dilutions was performed. Retina, ON, or superior colliculus sections were blocked in PBS-T-BSA for 1 h, then secondary antibodies (1:250; Alexa-Fluor 488, 594, or 647; Jackson ImmunoResearch, West Grove, PA, USA) prepared in PBS-T-BSA were added and incubated for 2 h. DAPI (4′,6-diamidino-2-phenylindole; 1:2000) was applied, then rinsed before sections were cover-slipped using Fluoromount-G (SouthernBiotech, Birmingham, AL, USA). Six to ten representative slides (four sections/slide) were imaged using a Leica DMi8 confocal microscope. We confirmed specificity by incubating sections with secondary antibodies but without primary antibodies. NeuroTrace, a Nissl stain selective for the Nissl substance characteristic of neurons (Emission wavelength 505, 1:100, Invitrogen, Waltham, MA, USA, N21480), was used on brain sections containing the superior colliculus. RGC counts in the ganglion cell layer (GCL) were obtained from sagittal sections that included the optic nerve and optic nerve head. RGC counts and tdT mean intensity in the regions of interest (retina, ON, and ONH) were analyzed using four sections per slide, and 5 slides per region using ImageJ. Ten sections of visual centers per slide per mouse were analyzed for tdT mean intensity.

### 2.12. Histopathology for Light Microscopy

ONs were post fixed in 2% PFA + 2% glutaraldehyde in 0.1 M sodium cacodylate buffer (CB, pH 7.4) for 48 h [7]. Briefly, tissues were incubated in 2% osmium tetroxide in 0.1 M cacodylate for 45 min after 3 washes in CB. Then, tissues were dehydrated through a series of graded alcohols followed by propylene oxide, then increasing concentrations of PolyBed resin culminated in final embedding and curing in PolyBed resin (Araldite 502/Polybed 812 kit, Polyscience, Inc, Warrington, PA, USA) in an oven at 60 °C. Using a Leica ultramicrotome, sections (500 nm) were cut, mounted on slides, then stained with 1% paraphenylenediamine (PPD) [7,16,17].

### 2.13. Quantification of Axons

Unbiased stereological analysis of axons in PPD-stained ON using a 100× objective was performed as previously described [7] using the optical fractionator module within StereoInvestigator (MicroBrightfield Bioscience, Williston, VT, USA). A 5 × 5 µm counting frame was utilized across roughly 40 sites. The coefficient of error (Schmitz-Hof) was maintained at 0.05 or below, ensuring sufficient sampling rate [7,18].

### 2.14. Protein Extraction for Capillary-Based Electrophoresis (WES)

Retina and myelinated ONs were collected in T-PER buffer with HALT protease and phosphatase inhibitors, then disrupted with a Branson Sonifier to create a protein lysate. After centrifugation for 15 min at 15,000 rpm, the supernatant was collected. Total protein concentration was measured by a Bicinchoninic Acid (BCA) assay kit. Proteins were analyzed by capillary tube-based electrophoresis immunoassay using the Wes and normalized to total protein in the sample. Wes is a Protein Simple instrument that separates proteins by an electrical charge in capillary tubes and allows binding of primary antibody then protein visualization within the capillary. Protein analysis for GLUT1 (1:50, rabbit, Novus Biologicals, Littleton, CO, USA, NB110-39113), GLUT3 (1:25, mouse, R & D systems, Minneapolis, MN, USA, NAB1415), HIF-1α (1:1000, mouse, Santa Cruz, Dallas, TX, USA, sc-13515), and HIF-2α (mouse, 1:1000, Santa Cruz, Dallas, TX, USA, sc-13596) was repeated at least three times with biological replicates [7,19].

### 2.15. Statistical Analysis

Statistical analysis was performed using GraphPad Prism 7 (GraphPad, La Jolla, CA, USA). Data were expressed as mean ± SEM and analyzed using unpaired, Student’s *t*-test (two groups) when comparing control versus experimental and one-way ANOVA followed by Tukey’s multiple-comparison post hoc test when making comparisons across more than two groups. The Kruskal–Wallis test was used to compare more than two groups and Dunn’s multiple comparison testing when a significant Bartlett’s test was detected. A *p*-value < 0.05 was considered statistically significant. Table 1 shows a breakdown of all of the sample numbers used in this study.

## 3. Results

### 3.1. Visually Evoked Potential Significantly Decreased after OHT

**Figure 1:**
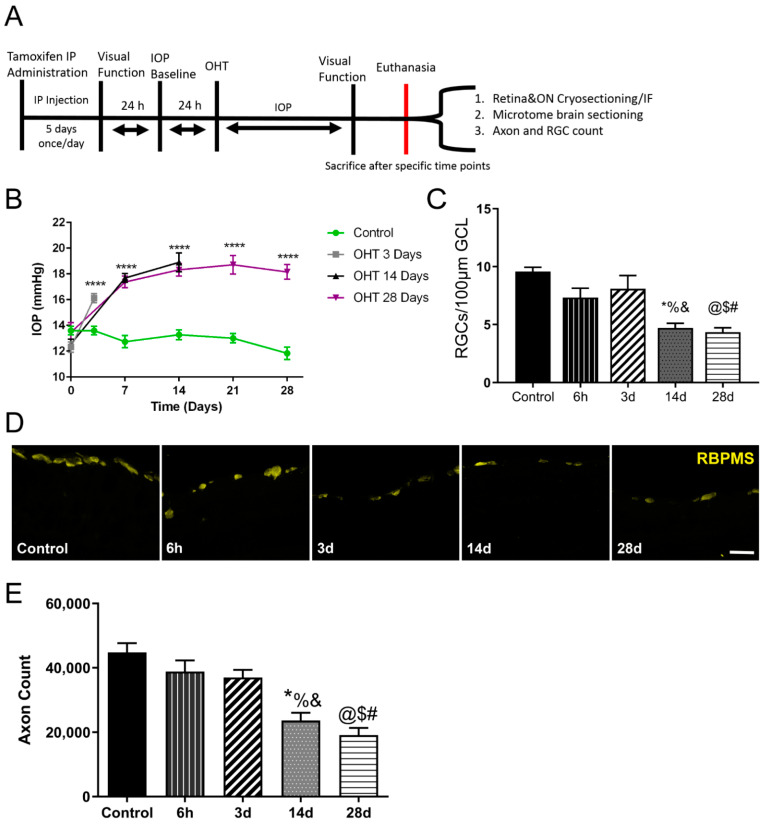
OHT results in increased IOP, RGC loss, and functional compromise. (**A**). Experimental design. Intraperitoneal injection of tamoxifen was administered to 2-month-old CAG-ODD transgenic mice. Visual evoked potential (VEP) and IOP were measured before and after 3, 14, and 28 days of OHT induction. OHT was induced by magnetic bead intracameral injection. Mice received an IP injection of 60 mg/kg pimonidazole, then were sacrificed 90 min later to compare and assess hypoxia using tdTomato (tdT) expression and pimonidazole (pimo) methods at 6 h, 3 days, 14 days, and 28 days after OHT in the retina, ON and visual centers in the brain. (**B)**. A significant IOP elevation was detected (****, *p* < 0.0001) 3, 14, and 28 d after OHT. n = 16 eyes at 3 d, 14 eyes at 14 d, 12 eyes at 28 d, and 30 control eyes. (**C**). A significant decrease (F _(4, 75)_ = 17.51) in RGC count per GCL was shown 14 d after OHT compared to control (%, *p* < 0.0001), 6 h (*, *p* < 0.0332), and 3 d (&, *p* = 0.0026). In addition, a significant decrease was detected in RGC count per µm GCL 28d after OHT compared to control (@, *p* < 0.0001), 6 h ($, *p* = 0.0052), and 3d (#, *p* = 0.0002); n = 12 eyes at 6 h, 12 eyes at 3 d, 15 eyes at 14 d, 21 eyes at 28 d, and 20 control eyes. (**D**). Representative examples of RGC immunofluorescence for the control, 6 h, 3 d, 14 d, and 28 d time points; scale bar = 25 µm. (**E**). A significant decrease (F _(4, 36)_ = 14.64) was shown in axon count 14d after OHT compared to control (%, *p* < 0.0001), 6 h (*, *p* = 0.0105), and 3 d (&, *p* < 0.0425). A significant decrease was also shown in axon count 28 d after OHT compared to control (@, *p* < 0.0001), 6 h ($, *p* = 0.0009), and 3 d (#, *p* = 0.0045); n = 7 ONs at 6 h, 6 ONs at 3 d, 8 ONs at 14 d, 7 ONs at 28 d, and 13 control ONs. Data represented combined results from recombinase negative (cre−) and recombinase positive (cre+) CAG-ODD transgenic mice. Days = d, and hours = h.

Figure 1A shows the experimental sequence of events for mice, including induction of cre recombinase expression using tamoxifen injection, visual function testing, induction of OHT, and sacrifice. There was no difference in IOP, RGC number, axon number, or VEP traces between the cre+ and cre− CAG-ODD mice, so cre+ and cre− mouse values were combined in Figure 1 panels. IOP was significantly elevated by 3 days after magnetic microbead injection to generate OHT in both cre− and cre+ CAG-ODD mice; this elevation was maintained for all mice subjected to OHT (Figure 1B). As expected, there was a significant decrease in RGC and axon number at 14 d and 28 d after OHT compared to control mice and mice in the 6h and 3d after OHT groups (Figure 1C,E). Figure 1D shows representative immunofluorescence for sections used to quantify RGCs. 

**Figure 2:**
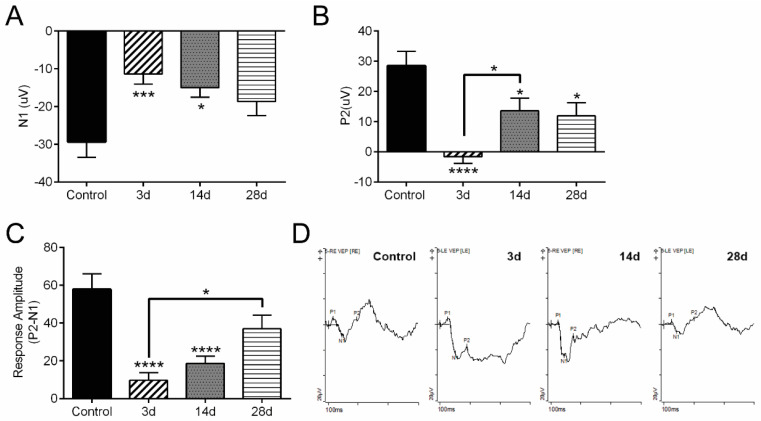
(**A**). A significant decrease (F _(3, 44)_ = 6.119) in N1 amplitude was observed after 3 d OHT (***, *p* = 0.0010) and 14 d OHT (*, *p* = 0.0111) compared to control. n = 14 eyes at 3 d, 14 eyes at 14 d, 8 eyes at 28 d, and 12 control eyes. (**B**). A significant decrease (F _(3, 44)_ = 10.60) in P2 amplitude 3 d OHT (****, *p* < 0.0001), 14 d OHT (*, *p* = 0.0375), and 28 d OHT (*, *p* = 0.0490) compared to control in addition to the significant decrease in P2 amplitude 3 d OHT compared to 14 d OHT (*, *p* = 0.0248). n = 14 eyes at 3 d, 14 eyes at 14 d, 8 eyes at 28 d, and 12 control eyes. (**C**). A significant decrease (F _(3, 44)_ = 14.38) in response amplitude was observed after 3 d OHT (****, *p* < 0.0001) and 14 d OHT (****, *p* < 0.0001) compared to control; in addition, the response amplitude at 3 d OHT was significantly lower than that at 28 d OHT (*, *p* = 0.0181). n = 14 eyes at 3 d, 14 eyes at 14 d, 8 eyes at 28 d, and 12 control eyes. (**D**). Representative VEP traces from mice prior to OHT (Control), then at 3 d, 14 d, and 28 d after OHT. Data represented combined results from recombinase negative (cre−) and recombinase positive (cre+) CAG-ODD transgenic mice. Days = d, and hours = h.

We analyzed VEP at 3, 14, and 28 d after hypoxia. A significant decrease in terminal VEP, including N1 and P2 amplitudes, was detected 3, 14, and 28 d after OHT in both cre− and cre+ CAG-ODD mice (Figure 2A,B). A significant decrease in P2 amplitude at 3 d compared to 14 d after OHT was detected. In addition, a significant decrease in terminal response amplitude (the amplitude of the N1 trough to P2 peak) was detected at 3, 14, and 28 d after OHT in both cre− and cre+ CAG-ODD mice (Figure 2C). Furthermore, the response amplitude at 3 d after OHT was significantly decreased compared to 28 d (Figure 2C), suggesting some recovery of VEP 28 d after OHT. Figure 2D shows representative VEP traces taken at baseline, prior to OHT (Control), then at 3 d, 14 d, and 28 d after OHT.

### 3.2. Hypoxic Glia and Neurons in Retina after OHT

**Figure 3:**
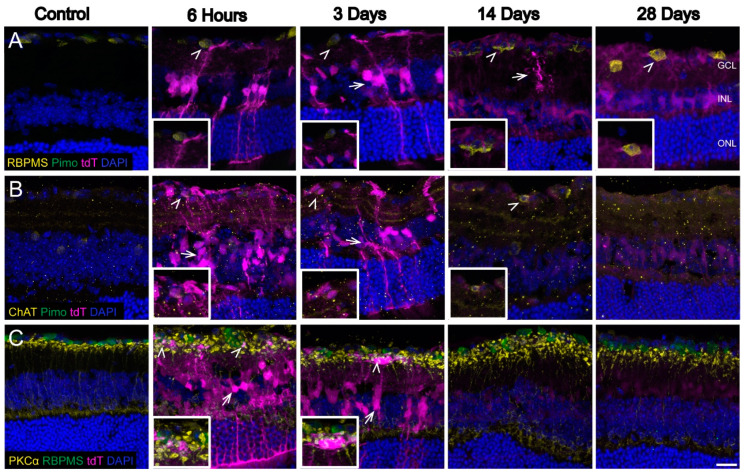
Hypoxic neurons in the retina after OHT. Sagittal retinal sections were labeled with neuronal markers (**A**). RBPMS (RNA binding protein multi-splicing, RGC marker-yellow, arrowhead) showed colocalization (arrowhead) with tdT in GCL. (**B**). ChAT (anti-choline acetyltransferase, amacrine cell marker, yellow) showed some colocalization with tdT. Arrows are pointing to tdT in Müller glia. Arrowhead is pointing at hypoxic amacrine cells at 6 h, 3 d, and 14 d. (**C**). PKCα (protein kinase c alpha, bipolar cell marker-yellow, arrowheads) showed some colocalization at 6 h and 3 d. RBPMS (shown in green in this panel). tdT (tdTomato) expression (magenta), DAPI (cell nuclei, blue). Retinal layers are labeled as follows: GCL (ganglion cell layer), INL (inner nuclear layer), and ONL (outer nuclear layer). Scale bar: 25 µm. n = 6 eyes at 6 h, 6 eyes at 3 d, 6 eyes at 14 d, 6 eyes at 28 d, and 10 control eyes. Insets represent a magnification of hypoxic neurons.

To determine which visual system cells showed evidence of hypoxia, we immunolabeled sagittal sections of retina from CAG-ODD mice subjected to OHT with antibodies against proteins that are specific for RGCs (RBPMS), subsets of amacrine cells (choline acetyltransferase, ChAT), and bipolar cells (PKCα) in Figure 3A–C, respectively. Although there was significant tdT labeling in the ganglion cell layer (GCL) at 6 h and 3 d after OHT (Figure 3A), the labeling corresponded to Müller cell endfeet. By 14 and 28 d, however, there was some colocalization of RBPMS with tdT (arrowhead at 6 h, 14 d, and 28 d). We observed colocalization of ChAT with tdT after OHT (Figure 3B). There was some colocalization of tdT with PKCα in bipolar cells at 6 h and 3 d after OHT (arrowheads, Figure 3C).

**Figure 4:**
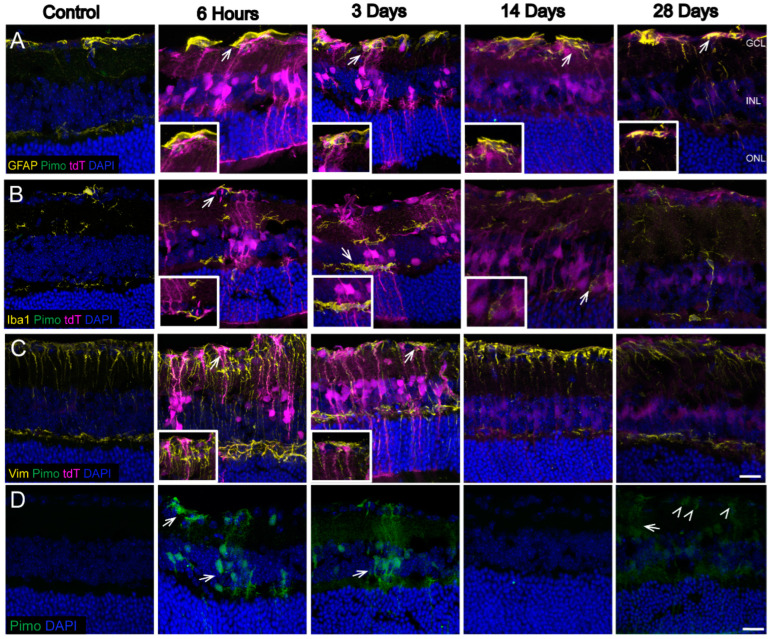
Hypoxic glia in the retina after OHT. Sagittal retinal sections were labeled with glial markers. (**A**). GFAP (glial fibrillary acidic protein, astrocyte marker, yellow) showed colocalization (arrows) with tdT (magenta) in GCL. (**B**). Iba-1 (ionized calcium binding adaptor molecule 1, microglia marker, yellow) showed colocalization (arrows) with tdT in INL. (**C**). Vim vimentin, Müller glia marker, yellow) showed colocalization (arrows) with tdT. (**D**). Pimonidazole (pimo, hypoxia marker, green, arrows) labeling was reported in Müller glia, and cells in GCL 6 h, 3 d, and 28 d after OHT. Pimonidazole was shown separately since it was overridden by tdT in previous images. tdT (tdTomato) expression (magenta), DAPI (cell nuclei, blue). Retinal layers were labeled as follows: GCL (ganglion cell layer), INL (inner nuclear layer), and ONL (outer nuclear layer). Scale bar: 25 µm. n = 6 eyes at 6 h, 6 eyes at 3 d, 6 eyes at 14 d, 6 eyes at 28 d, and 10 control eyes. Insets represent a magnification of hypoxic glia.

In Figure 4, immunolabeling with antibodies against proteins specific for astrocytes and Müller glia (GFAP), microglia (Iba1), or Müller glia (Vimentin) was undertaken to determine which glial cell types were exhibiting the tdT label. The majority of tdT labeling, denoting HIF-1α stabilization and thus, hypoxia, occurred in Müller glia after OHT. The hypoxia labeling was evident by 6h and remained strong at 3 d after OHT across all analyzed retinal sections. By 14 and 28 d after OHT, the tdT had dissipated to a degree, though it could still be observed in the ganglion cell layer (GCL) and the inner nuclear layer (INL), corresponding to the location for Müller glia cell bodies. Labeling for tdT also colocalized with GFAP, Vimentin (Vim) and Iba1 (see arrows in Figure 4A–C).

To compare the specificity of the CAG-ODD construct, we also injected some mice with pimonidazole, a chemical that forms covalent adducts on proteins in cells exposed to hypoxic conditions (<10 mm Hg oxygen), see Figure 4D. Pimonidazole was detected in astrocytes, Müller glia (arrows), and RGCs or amacrine cells (arrowheads) 6 h, 3 d, and 28 d after OHT (Figure 4D), a finding that corroborates our previous study [7]. Pimonidazole was shown separately because it was otherwise masked by the intense labeling of tdT. Overall, the pimonidazole labeling was observed in the same cell types as detected by the tdT but was not as widespread.

### 3.3. Strong Hypoxia in Optic Nerve (ON) Glia after OHT

**Figure 5:**
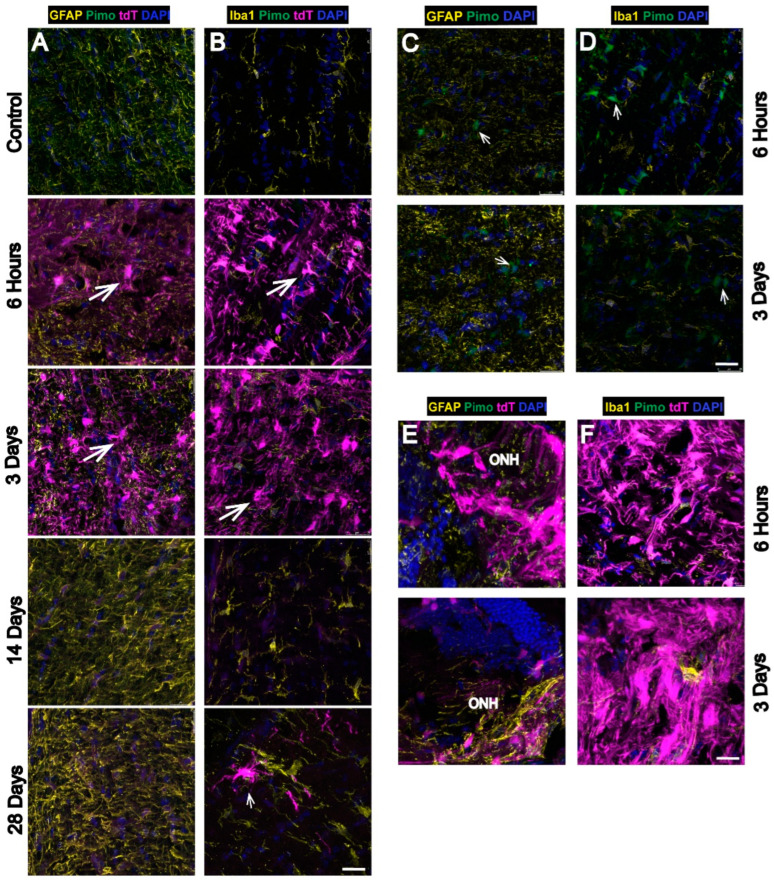
Hypoxic glia in the ON and ONH after OHT. Sections oriented so the globe is superior. Longitudinal sections of ON showed hypoxic glia detected 6 h, 3 d, 28 d after OHT by tdT expression colocalized with GFAP (yellow) in astrocytes ((**A**), arrows) and Iba-1 (yellow) in microglia ((**B**), arrow) compared with control. Pimonidazole-positive glial cells (pimo, green, arrows) were shown colocalized with GFAP in astrocytes (**C**) and with Iba1 in microglia (**D**) 6 h and 3 days after OHT. Pimonidazole was shown separately since tdT expression overrode pimonidazole labeling in **A**,**B**. ONH showed higher intensity of tdT expression than ON 6hrs and 3d after OHT, which colocalized with GFAP in astrocytes (**E**) and with Iba-1 in microglia (**F**). ON: optic nerve, and ONH: optic nerve head. Pimonidazole (pimo, green), tdT = tdT expression (magenta), DAPI (cell nuclei, blue). Scale bar: 25 µm. n = 6 ONHs and ONs at 6 h, 6 ONHs and ONs at 3 d, 6 ONHs and ONs at 14 d, 6 ONHs and ONs at 28 d, and 6 control ONHs and ONs.

Like the retina, tdT-positive cells were most prominent at 6 h and 3 d after OHT in the myelinated ON. Cells positive for tdT colocalized with GFAP (astrocytes; Figure 5A) and Iba1 (microglia; Figure 5B). Though tdT was significantly decreased at 14 and 28 d compared to 6 h and 3 d after OHT, the occasional cell with strong labeling can still be observed (see Iba1 panel at 28 d, Figure 5B). The tdT labeling at 28 d after OHT in the ON was not observed using pimonidazole [7], suggesting that the CAG-ODD hypoxia reporter strategy is more sensitive than pimonidazole labeling. Pimonidazole-positive microglia and astrocytes were evident after 6 h and 3 d of OHT (Figure 5C,D) but did not label hypoxic astrocytes or microglia 14 and 28 d after OHT (data not shown).

### 3.4. Hypoxia Is Highest in the Optic Nerve Head (ONH) after OHT

The ONH showed the highest intensity of tdT expression 6 h and 3 d after OHT (Figure 5E,F) compared to control. The 14 and 28 d time points are not shown since they are comparable to the ON at 6 h and 3 d. The tdT colocalized with both GFAP and Iba1, though it also appeared to exceed the cumulative labeling of each.

**Figure 6:**
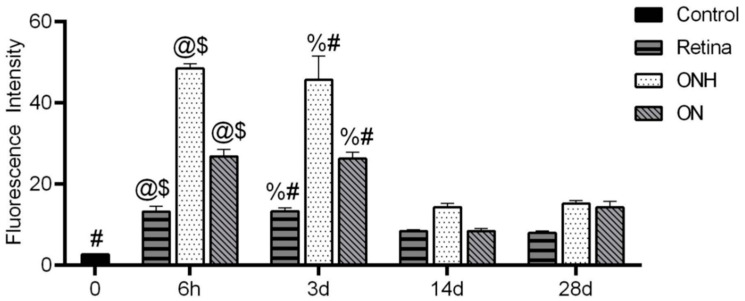
Hypoxia is highest in ONH and ON compared to retina. ON, ONH, and retina at each time point (6 h, 3 d, 14 d, and 28 d) showed significant tdT intensity compared to control (each timepoint and structure vs. baseline (#, *p* < 0.0001 except the following time points compared to control: 14 d retina (*p* = 0.0328), 28 d ONH (*p* = 0.0017), 14 d ONH (*p* = 0.0030), and 28 d ONH (*p* = 0.0010). ONH and retina tdT fluorescence intensity is significantly greater at each of the 6 h and 3 d time points as compared to the 14 d and 28 d time points (@$, *p* = 0.0001). ON tdT fluorescence intensity is significantly greater at 6 h compared to 14 d (@, *p* < 0.0001) and 28 d ($, *p* = 0.001) and is also greater at 3 d compared to 14 d (%, *p* < 0.0001) and 28 d (#, *p* = 0.0008). n = 6 ONHs and ONs at 6 h, 6 ONHs and ONs at 3 d, 6 ONHs and ONs at 14 d, 6 ONHs and ONs at 28 d, and 6 control ONHs and ONs.

Figure 6 shows the quantification of tdT labeling intensity in the ON, retina and the ONH. When compared across the 6 h and 3 d time points after OHT, the ONH had significantly stronger tdT labeling than the ON and the retina. Within the ON, tdT mean intensity was significantly greater than control at 6 h and 3 d, and 28 d after OHT. After a small dip in tdT intensity at 14 d, the hypoxia label increased, although non significantly (*p* = 0.064), at 28 d compared to 14 d after OHT. In the ONH, all time points showed a significant increase in tdT mean intensity compared to control. The 6 h and 3 d after OHT time points had significantly greater tdT mean intensity than 14 and 28 d after OHT. Within the retina, all time points showed a significant increase in tdT mean intensity compared to control. A significantly greater tdT mean intensity was detected 6 h and 3 d after OHT compared to 14 and 28 d after OHT.

### 3.5. Hypoxia in Brain Visual Nuclei after OHT

**Figure 7:**
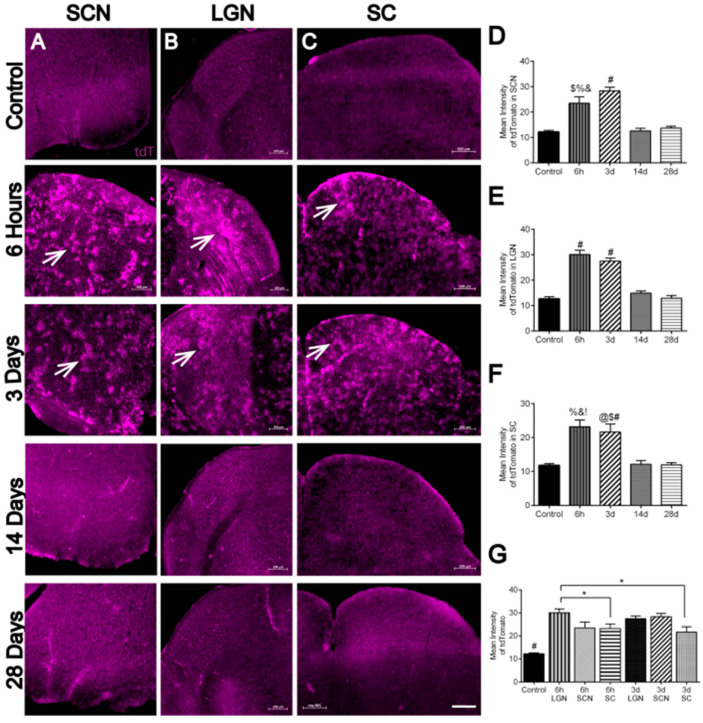
Hypoxia evident in the visual centers in the brain after OHT. Coronal sections of the brain showed significant hypoxia (tdT: tdTomato expression, magenta, arrows) in the SCN (**A**), LGN (**B**), and SC (**C**) 6 h, and 3 d after OHT compared to control and some td Tomato expression 14 and 28d after OHT. Scale bar: 200 µm. (**D**). A significant increase (F _(4, 16)_ = 28.15) in tdT was detected in glaucomatous SCN 6 h after OHT compared to control ($, *p* = 0.0005), 14 d (%, *p* = 0.0007), and 28 d (&, *p* = 0.0019) after OHT. A significant increase in tdT was shown in glaucomatous SCN 3 d after OHT compared to control, 14 d, and 28 d after OHT (#, *p* < 0.0001). n = 4 SCNs at 6 h, 5 SCNs at 3 d, 4 SCNs at 14 d, 4 SCNs at 28 d, and 4 control SCNs. (**E**). A significant increase (F _(4, 16)_ = 52.63) in tdT was detected in glaucomatous LGN 6 h and 3 d after OHT compared to control, 14 d, and 28 d after OHT (#, *p* < 0.0001). n = 4 LGNs at 6 h, 4 LGNs at 3 d, 4 LGNs at 14 d, 5 LGNs at 28 d, and 4 control LGNs. (**F**). A significant increase (F _(4, 17)_ = 14.79) in tdT was shown in glaucomatous SC 6 h after OHT compared to control (%, *p* =0.0005), 14 d (&, *p* = 0.0006), and 28 d (!, *p* = 0.0003) after OHT. A significant increase in tdT was shown in glaucomatous SC 3 d after OHT compared to control (@, *p* = 0.0033), 14 d ($, *p* = 0.0044), and 28 d (#, *p* = 0.0023) after OHT. n = 5 SCs at 6 h, 4 SCs at 3 d, 4 SCs at 14 d, 5 SCs at 28 d, and 4 control SCs. (**G**). A significant increase (F _(6, 31)_ = 27.26) in tdT mean intensity was evident in SCN, LGN, and SC 6 h, 3 d, 14 d, and 28 d after OHT compared with the control (#, *p* < 0.0001). A significant increase in tdT mean intensity was shown 6h after OHT in LGN compared to 6 h (*, *p* = 0.0499), and 3 d OHT in the SC (*, *p* = 0.0143). SCN = suprachiasmatic nucleus, LGN = lateral geniculate nucleus, and SC = superior colliculus.

We examined retino-recipient areas of the brain, including the suprachiasmatic nucleus (SCN), lateral geniculate nucleus (LGN), and superior colliculus (SC), for tdT labeling after OHT. A significant increase in tdT expression was evident at 6 h and 3 d after OHT in the SCN (Figure 7A,D) and LGN (Figure 7B,E). Similarly, at 6 h and 3 d after OHT, there was a significant increase in tdT labeling in the SC compared to control, 14 d, and 28 d (Figure 7C,F). We quantified the intensity of tdT labeling, finding significantly higher tdT in the LGN 6h after OHT compared to the SC at both 6 h and 3 d after OHT (Figure 7G).

**Figure 8:**
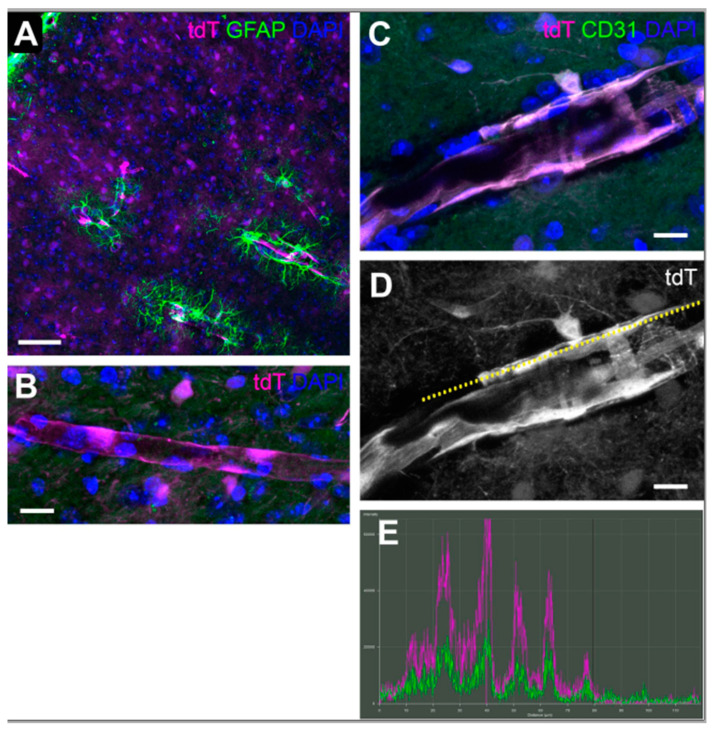
Collicular vasculature is hypoxic 6 h after OHT. (**A**). Immunofluorescence for GFAP (green) in superior colliculus 6h after OHT shows vasculature positive for tdTomato (magenta), indicating ODD stabilization from hypoxia. Cell nuclei labeled with DAPI (blue). 20× magnification; Scale bar = 100 µm. (**B**). A collicular vessel positive for tdTomato, 60× magnification. Scale bar = 20 µm. (**C**). A larger vessel in the superior colliculus labeled with tdTomato (magenta), endothelial marker CD-31 (green), and nuclear stain DAPI (blue). 60× magnification. Scale bar = 20 µm. (**D**). The magenta labeling (alone in grayscale) for the same vessel as (**C**) with a dotted line (yellow) to show the structure drawn to evaluate colocalization of tdTomato with CD-31; results shown in (**E**). (**E**). Colocalization plot for the yellow dotted line in (**D**); magenta is tdTomato and green is CD-31. Peak correspondence indicated colocalization of the two fluorophores within the vessel. Five brains per group and ten sections per brain were analyzed from the 4 time points (6 h, 3 d, 14 d, 28 d).

The superior colliculus contains the RGC synapses furthest removed from the retina, so we determined revealing the cell types that were positive for the tdT hypoxia reporter there. The tdT label was observed in the collicular vasculature, surrounded by GFAP-positive astrocyte endfeet (Figure 8A), or alone (Figure 8B). Figure 8C–E show a vessel at high magnification labeled with endothelial marker CD-31; in Figure 8D, a yellow dotted line shows the placement of the colocalization plot line, with results in Figure 8E. The CD-31 immunolabel is colocalized with the tdTomato hypoxia reporter, as shown by the coincidence of green (CD-31) and magenta (tdT) labeling peaks (Figure 8E).

**Figure 9:**
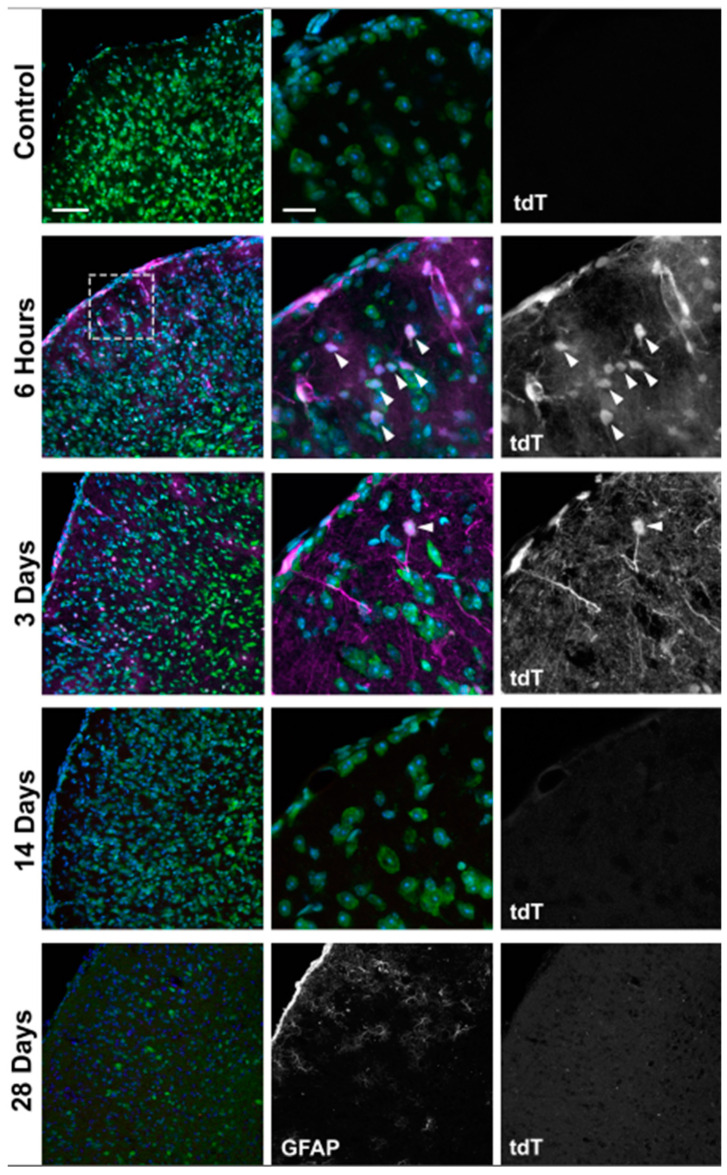
A timecourse of superior colliculus sections after OHT. Superior colliculus was stained using NeuroTrace (green) and DAPI (blue); tdTomato (tdT, magenta) reporter indicates cells subjected to hypoxia. Left panels were imaged at 20× magnification (scale bar = 50 µm) and center panels at 60× magnification (scale bar = 20 µm). Right panels are the tdTomato fluorescence alone, in grayscale. In the control superior colliculus, no tdTomato label (right panel) was observed. tdTomato labeling was most widespread at 6 h and 3 d after OHT, including colocalization with NeuroTrace-positive cells (arrowheads). The dotted line in the 6 h panel is the outline of the 60× center panel. By 14 d, tdTomato labeling was no longer evident in the colliculus. In the 28 d panels, all are at 20× magnification, with the center showing GFAP labeling and the right the tdT. Five brains per group and ten sections per brain were analyzed from the 4 time points (6 h, 3 d, 14 d, 28 d).

We used NeuroTrace to label neurons in the superior colliculus sections, finding at 6 h and 3 d after OHT that neurons were positive for tdT label (Figure 9, arrowheads). Hypoxia reporter label was barely detectable by 14 and 28 d after OHT (Figure 9, 14 d and 28 d panels). No tdT label was observed in the non-OHT (Control) superior colliculus.

### 3.6. Significant Changes in GLUT1 and GLUT3 Levels after OHT

**Figure 10:**
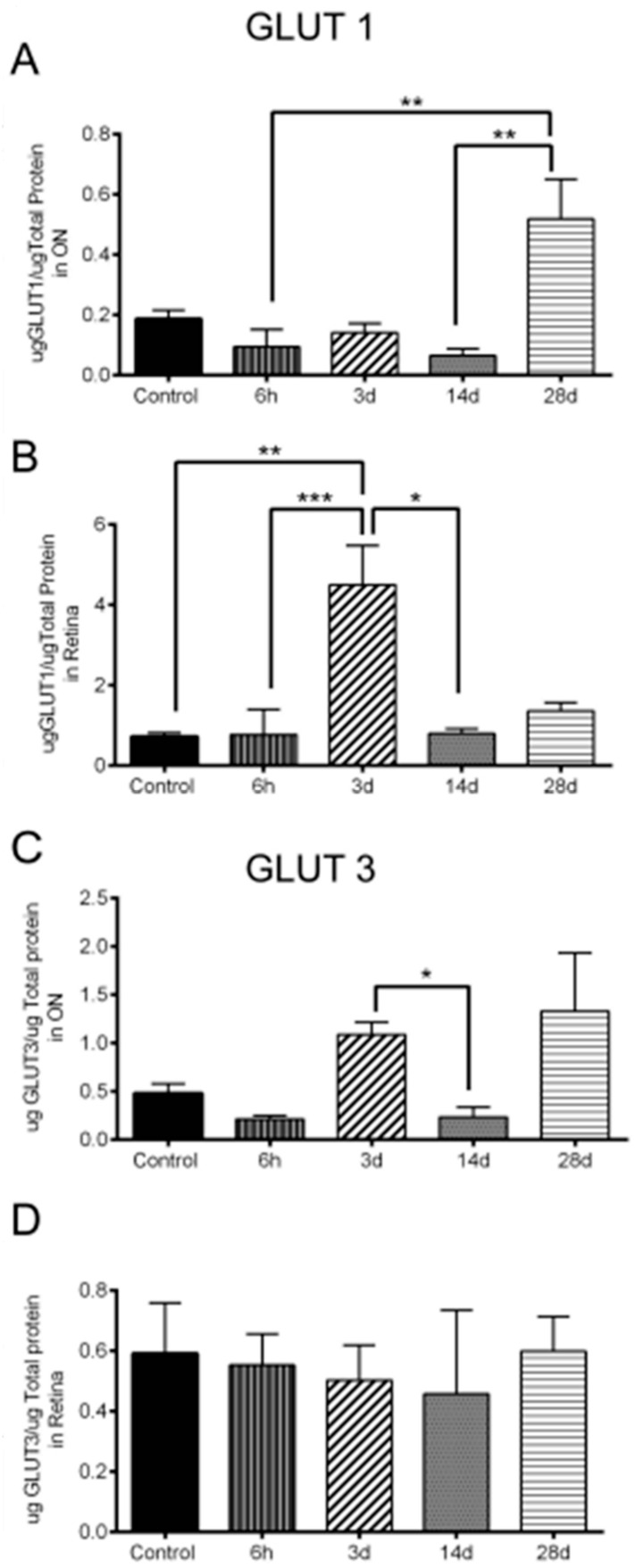
Significant changes in GLUT1 and GLUT3 levels after OHT. (**A**). Significant GLUT1 level increase in the ON 28 d after OHT compared to 6 h (**, *p* = 0.0094), and 14 d (**, *p* = 0.0059). n = 6 ONs at 6 h, 7 ONs at 3 d, 6 ONs at 14 d, 4 ONs at 28 d, and 18 control ONs. (**B**) Significant GLUT1 level increase in the retina 3 d after OHT compared to control (**, *p* = 0.0039), 6 h (***, *p* = 0.0008), and 14 d (*, *p* = 0.0488). n = 6 retinas at 6 h, 6 retinas at 3 d, 6 retinas at 14 d, 4 retinas at 28 d, and 18 control retinas. (**C**). Significant GLUT3 level increase in the ON 3 d after OHT compared to 14 d (*, *p* = 0.0426). n = 5 ONs at 6 h, 7 ONs at 3 d, 6 ONs at 14 d, 5 ONs at 28 d, and 19 control ONs. (**D**). No significant change in GLUT3 level in the retina 6 h, 3 d, 14 d, and 28 d after OHT (*p* > 0.05). n = 9 retinas at 6 h, 11 retinas at 3 d, 6 retinas at 14 d, 4 retinas at 28 d, and 18 control retinas.

While GLUT1 protein significantly increased 28 d after OHT in ON compared to 6 h and 14 d after OHT, it significantly increased in the retina 3 d after OHT compared to control, 6 h, and 28 d (Figure 10A,B). GLUT3 protein, on the other hand, significantly increased in the ON 3 d after OHT compared to 14 d; there was no change in GLUT3 of the retina at any time point (Figure 10C,D). See supplementary data of original electrophoresis blots, see Figure S1. 

Mice that were cre-recombinase negative (cre−) yielded results comparable to control; there was no tdT expression after OHT. Therefore, we did not show cre− animal results.

## 4. Discussion

We determined the timeline of hypoxia after OHT in the visual pathway using a transgenic hypoxia reporter mouse. Hypoxia commenced with high intensity tdT expression at 6 h and 3 d after OHT in the entire visual pathway: Retina, ON, ONH, and visual centers in the brain. The highest intensity of hypoxia reporter labeling was observed in the ONH. By 14 d and 28 d after OHT, the hypoxia reporter was significantly decreased compared to 6 h and 3 d in the visual pathway; however, it did not return to control levels. Hypoxic glia (microglia, astrocytes, Müller glia) and, to a much lesser extent, neurons (RGCs, amacrine cells, bipolar cells), were evident in the retina. In the ON and ONH, hypoxic GFAP-positive astrocytes and Iba1-positive microglia were observed. Müller glia were the cells most hypoxic after OHT, as measured by tdT labeling in the retina. In the superior colliculus, endothelial cells and neurons were observed to be hypoxic. Overall, hypoxia was more evident in glial cells compared to neurons, suggesting a hypoxia management role for glia.

We observed hypoxia in the retina, ON, and superior colliculus within 6h of OHT using the microbead model. Thus, the hypoxia-driven tdTomato indicates that ocular blood flow was reduced within 6 h of OHT induction. The early timing of this hypoxia suggests an initiating IOP spike with bead injection since the IOP measured at 3 d was 16 mm Hg, only 4 mm Hg higher than baseline. Due to the relatively slow onset and chronic nature of primary open angle glaucoma in human patients, there is an expectation that hypoxia/ischemia, and thus, IOP spiking, does not reliably model glaucoma. However, data from patients and non-human primates indicates IOP can spike up to 10 mm Hg from eyeblink, up to 90 mm Hg from squeezing one’s eyelids shut [20], or even as a result of acute stress [21]. These IOP changes, though relatively short-lived, are nonetheless capable of reducing ocular bloodflow to a degree that can lead to intermittent hypoxia/ischemia [22]. We also do not yet understand the impact of potentially thousands of IOP spikes daily, particularly for individuals susceptible to developing glaucoma. Significantly, the instability of oxygen supply to the retina or optic nerve through changes in IOP may further exacerbate glaucoma-associated optic neuropathy in individuals with disturbed autoregulation of ocular blood flow [23], a leading hypothesis for the mechanism of normal tension glaucoma. Additional work is necessary to establish how often and to what degree hypoxia contributes to glaucomatous neurodegeneration, but the evidence for IOP spiking in patients and in animal models suggests that it is of value to examine its impact. In research from the Pardue lab, a significant decline in the b-wave amplitude of the flash electroretinogram indicated whether a retina had been exposed to ischemia [24]. We evaluated VEP in our study, not flash ERG, so cannot apply this yardstick to our data. The early onset of hypoxia (6 h) does suggest an IOP spike with OHT induction. However, the maintenance of tdTomato labeling at 3 d, beyond the point at which the tdTomato protein would have turned over [25], suggests that hypoxia induction extended beyond the induction of OHT.

Visual function measured by VEP significantly decreased 3 d after OHT, which coincided with initial IOP elevation. However, IOP remained elevated through the 28 d of OHT while the VEP response amplitude gradually increased, though it never achieved control levels. The improvement of visual function seen 28 d after OHT may be the result of compensation from functional RGCs. The minimum number of RGCs needed to achieve visual function is still unknown. Interestingly, Park et al. have found that after induced IOP elevation in rats, presynaptic and postsynaptic vesicle proteins increased between RGCs and bipolar cells. This finding indicates that the loss of RGCs may drive new synaptic contact formation between RGCs and bipolar cells [26], potentially relieving the initial loss of synapses. In addition, the significant increase in GLUT1 level 28 d after OHT in the ON could have increased glycolysis and preserved some axons, which contributed to better visual function.

These findings confirm the impact of IOP elevation on initiating damage to RGCs and their axons but also suggest a potential functional adaptation to elevated IOP. The greatest decrement in visual function corresponded to the most intense hypoxia reporter labeling (3 d after OHT), suggesting that OHT-induced hypoxia contributes significantly to visual system dysfunction. These alterations occur prior to the significant declines in RGC and axon numbers at 14 and 28 d after OHT, which further supports that early hypoxia can drive glaucoma progression.

### 4.1. Hypoxic Glia and Neurons in Glaucomatous Retina

The OHT retina showed hypoxic microglia, astrocytes, Müller glia, bipolar cells, amacrine cells, and RGCs, but primarily Müller glia. In all cases, cells showed the highest labeling early (6 h and 3 d) after OHT but continued with lesser intensity at 14 and 28 d after OHT. The visual structures evaluated always showed minor hypoxia in glial cells, even out to 28 d, never returning to control levels. Reporter expression is a result of stabilization of the ODD domain of HIF protein. Interestingly, our previous publication found a significant increase in HIF-1α protein at 28 d compared to 14 d in the retina [7], despite our observation here that tdT reporter labeling remained relatively flat at 14 and 28 d, though higher than control. One explanation could be the masking of individual cells’ hypoxia response in whole retina lysate that was used to measure HIF-1α in the previous publication [7]. In support of that, it was more often the case that we observed stand-out individual cell tdT labeling at 28 d than at 14 d (for example, microglia in the ON as shown in Figure 5B). Small numbers of individual cells with intense tdT labeling would not have appreciably altered total retinal HIF-1α levels.

Here, we found some evidence of hypoxia in RGCs, bipolar cells, and amacrine cells. There have been few cell-specific examinations of hypoxia in the retina during glaucoma. In an oxygen-induced retinopathy mouse model, HIF-1α staining was prominent in cells across the inner nuclear layer (INL) and ganglion cell layer (GCL), whereas HIF-2α was highly restricted to Müller glia and astrocytes [10]. In addition, HIF-2α was expressed at a higher level than HIF-1α within the hypoxic inner retina. We have observed activation of HIF-1α but no change in HIF-2α protein in the retina and ON after OHT [7]; however, we did not use immunolabeling to distinguish among cell types at the time. Further investigation is warranted to unravel the cellular specificity of HIF-1α and HIF-2α.

Since Müller glia span the entire retina, they may be exposed to stress more dramatically than other retinal cells. Müller glia became activated as early as 2–3 days after OHT in rats as determined by upregulation of GFAP; the activation was sustained for several months [27,28]. The dramatic HIF stabilization that occurred in Müller glia in our study suggests that these cells are quite sensitive to oxygen levels. During hypoxia, metabolic activity is converted from aerobic respiration, oxidative phosphorylation (OxPhos), to anaerobic glycolysis by hypoxia-activated, HIF-1-mediated transcriptional activity, which suppresses mitochondrial aerobic metabolic processes, including the tricarboxylic acid cycle and OxPhos [29,30,31]. Müller glia have a reputation for being glycolytic cells because they secrete lactate [32]. Müller glia are also capable of maintaining ATP production during complex III inhibition; this was interpreted to mean Müller glia are preferentially glycolytic as well [33]. However, Müller glia have significant mitochondria in their basal processes [34], and low levels of pyruvate kinase [35] that suggest an impaired ability for glycolysis. There is evidence that Müller glia utilize lactate released from photoreceptors to generate ATP through mitochondria [35]. The sensitivity of Müller glia for oxygen. as shown through tdT labeling, would support a Müller glial need for oxygen and potentially a preference for OxPhos. It is not clear if HIF activation could propel Müller glia into a state more supportive of neuronal survival because hypoxic Müller glia and neurons undergo a HIF-activated transition to glycolysis that would increase competition for the available glucose [32,36].

Reduced oxygenation during hypoxia stabilizes HIF-α, which promotes the upregulation of glucose transporters (GLUTs) to induce glycolysis and maintain ATP availability, enabling metabolic adaptation to limited oxygen [11]. We found a significant increase in *glut1* (astrocytic) transcripts [7], and GLUT1 protein 3 d after OHT in the retina compared to control, 6 h, and 14 d. In ON, GLUT1 protein was low 14 d after OHT, timing that matches our detection of no tdT expression 14 d after OHT in ON. However, GLUT1 in ON at 28 d was higher than at any other time point. This raises the possibility that, in the ON, alterations to improve glucose transport can be delayed when compared to the timing of the impetus for that increase (hypoxia). In contrast to the astrocyte-associated glucose transporter, *Glut3* (neuronal) mRNA, but not protein, significantly increased in glaucomatous DBA/2J ON [37]. Similarly, GLUT3 protein did not change at all after OHT in the retina. However, in the ON, GLUT3 significantly increased 3 d after OHT (compared to 14 d), a time frame that would suggest that the upregulation was a response to the hypoxia occurring there. IOP elevation compromises blood flow to the visual pathway and that leads to oxygen and glucose deprivation. This glucose deprivation will drive the use of glycogen stores in cells, but glycogen would be depleted after prolonged hypoxia, therefore leading to metabolic vulnerability. Indeed, GLUT could increase at 3 d in the ON, but if glucose is not also available, then there will be no glycolysis nor subsequent ATP production. There were fewer hypoxic RGCs compared to glia early after OHT (6 h and 3 d). However, tdT expression seems to be more localized in cells, specially RGCs, later after OHT (14 and 28 d), which is coincident with RGC degeneration. This suggests that hypoxia may contribute to RGC degeneration.

### 4.2. Hypoxic Astrocytes and Microglia in Glaucomatous ON with the Highest Hypoxia in ONH

Surprisingly, our current and recently [7] reported data for the ON showed an absence of hypoxic cells at 14d but, although nonsignificant, their presence 28 d after OHT (Figure 5B). One cell type, microglia, stood out for late tdT labeling in the ON. We show hypoxic microglia at each of the time points analyzed, and in the retina, ONH, and ON. RGC death is induced by microglial-derived pro-inflammatory cytokines during neonatal hypoxia [38]. It is likely that the hypoxia response in microglia shown here also contributes to inflammation and facilitates RGC loss and dysfunction as a result of neuronal-microglia interaction. Indeed, microglia make brief and direct contact with neuronal synapses [39]. Reduced neuronal activity reduces microglia-neuron contact frequency. Prolonged microglia–synapse contacts are observed after transient cerebral ischemia followed by the disappearance of synapses, suggesting microglia contribution to synaptic disconnection [40]. Kaur et al. showed that hypoxia-induced microglia activation in the developing periventricular white matter, cerebellum and the retina. Activated microglia released TNF-α and IL-1β, which was coupled with NO, iron, and ROS accumulation, which collectively resulted in neuroinflammation and death of oligodendrocytes, Purkinje neurons and the RGCs [41].

Mitochondrial density is higher in the unmyelinated axons in the prelaminar and laminar optic nerve to meet the high energy demand for electrical conduction [42]. Therefore, we expect that the ONH may be more susceptible to the mitochondrial dysfunction [43,44] and metabolic vulnerability [37] observed in glaucoma. Our ONH findings support that the ONH has the greatest sensitivity toward IOP elevation because of the significant hypoxia response recorded in the astrocytes of the glial lamina and the microglia. After laser photocoagulation of the trabecular meshwork, Chidlow et al. reported that the ONH is the pivotal site of RGC injury, as protein accumulation was detected within ONH axons 8 h after OHT. In addition, axonal cytoskeletal damage was detected after 3 d and later time points following OHT [45].

### 4.3. Hypoxia in Visual Centers of the Glaucomatous Brain

The glaucomatous visual centers in the brain (SCN, LGN, SC) showed a significant increase in hypoxia 6 h and 3 d after OHT. Hypoxia was evident in these visual centers at the same time points hypoxia was most significantly detected in the retina, ON, and ONH. Since the visual centers do not experience IOP increase directly, the tdT reporter expression observed there may be the result of oxygen depletion through the visual pathway, possibly through changes in bloodflow. RGC projections may have become a sink for any available oxygen with the advent of hypoxia. In support of this, we observed that tdT mean intensity peaked first in the LGN, followed by the SC, the most distal portion of the RGC axon projection. Although visual dysfunction and increased HIF-1α stabilization persist, hypoxia was not as profound 14 and 28 d after OHT because visual centers were devoid of tdT expression. At both 6 h and 3 d, endothelial cells and NeuroTrace-positive neurons in the superior colliculus carried the tdT label.

Despite ramified microglia being dependent on OxPhos [46,47], we observed no stabilization of HIF in microglia in the retinorecipient regions in the brain.

### 4.4. The CAG-ODD Reporter System

We found that tdT expression upon cre-recombinase activation was a more sensitive approach to detecting hypoxia after OHT than an intraperitoneal injection of pimonidazole, the compound that forms covalent adducts in cells that have a partial pressure of oxygen less than 10 mm Hg. There were more cell types and time points in which the CAG-ODD reporter was observed compared to pimonidazole. For example, significant tdT labeling intensity was observed at 14 d in the retina and ONH, whereas no pimonidazole labeling was observed in these structures at 14 d. Additionally, there was tdT expression in hypoxic microglia 28 d after OHT in ON while no pimondazole-positive cells were detected. These results, compared to our recent findings using pimonidazole, indicate that tdT expression is a more sensitive method [7]. Importantly, tdT is a short-lived protein, degrading within 24 h [25]. Relatively fast tdT turnover confirms that tdT labeling we observed at 3, 14, and 28 d after OHT is a result of new reporter expression from recent hypoxia and does not represent the leftover tdT label. Despite the ubiquity and strength of the CAG reporter, its expression will nevertheless vary across cell types, which could conceivably affect reporter expression.

This study provides evidence for pre-degenerative hypoxia in the visual pathway that may drive glaucoma progression; therefore, hypoxia represents a potential therapeutic target. Hypoxia and visual dysfunction following IOP elevation were evident in the first 6 h and out to 28 d. The impact of events downstream of hypoxia response, such as metabolic disruption and glial activation, are likely prime contributors to pathology in the retina, ONH, ON, and visual centers of the brain. The profound hypoxia response in Müller glia suggests greater attention should be paid to their mechanisms of hypoxia management.

## Figures and Tables

**Table 1:** Sample numbers by experimental figure.

Figure	Experiment	Eye/ON Number
1/B	IOP measurement	n = 16 eyes at 3 d, 14 eyes at 14 d, 12 eyes at 28 d, and 30 control eyes
1/C	RGC count	n = 12 eyes at 6 h, 12 eyes at 3 d, 15 eyes at 14 d, 21 eyes at 28 d, and 20 control eyes.
1/E	Axon count	n = 7 ONs at 6 h, 6 ONs at 3 d, 8 ONs at 14 d, 7 ONs at 28 d, and 13 control ONs.
2/A	N1 amplitude	n = 14 eyes at 3 d, 14 eyes at 14 d, 8 eyes at 28 d, and 12 control eyes.
2/B	P2 amplitude	n = 14 eyes at 3 d, 14 eyes at 14 d, 8 eyes at 28 d, and 12 control eyes
2/C	Response amplitude	n = 14 eyes at 3 d, 14 eyes at 14 d, 8 eyes at 28 d, and 12 control eyes.
3	Retinal hypoxic neurons	n = 6 eyes at 6 h, 6 eyes at 3 d, 6 eyes at 14 d, 6 eyes at 28 d, and 10 control eyes
4	Retinal hypoxic glia	n = 6 eyes at 6 h, 6 eyes at 3 d, 6 eyes at 14 d, 6 eyes at 28 d, and 10 control eyes
5	Hypoxic glia in ON/ONH	n = 6 ONHs and ONs at 6 h, 6 ONHs and ONs at 3 d, 6 ONHs and ONs at 14 d, 6 ONHs and ONs at 28 d, and 6 control ONHs and ONs
6	Highest hypoxia in ONH/ON	n = 6 ONHs and ONs at 6 h, 6 ONHs and ONs at 3 d, 6 ONHs and ONs at 14 d, 6 ONHs and ONs at 28 d, and 6 control ONHs and ONs
7/A–D	Hypoxia in SCN	n = 4 SCNs at 6 h, 5 SCNs at 3 d, 4 SCNs at 14 d, 4 SCNs at 28 d, and 4 control SCNs.
7/E	Hypoxia in LGN	n = 4 LGNs at 6 h, 4 LGNs at 3 d, 4 LGNs at 14 d, 5 LGNs at 28 d, and 4 control LGNs
7/F	Hypoxia in SC	n = 5 SCs at 6 h, 4 SCs at 3 d, 4 SCs at 14 d, 5 SCs at 28 d, and 4 control SCs
8	Hypoxia in SC	n = 5 SCs at 6 h, 3 d, 14 d, 28 d; 10 sections per SC
9	Hypoxia in SC	n = 5 SCs at 6 h, 3 d, 14 d, 28 d; 10 sections per SC
10/A	GLUT1 increase in ON	n = 6 ONs at 6 h, 7 ONs at 3 d, 6 ONs at 14 d, 4 ONs at 28 d, and 18 control ONs
10/B	GLUT1 increase in retina	n = 6 retinas at 6 h, 6 retinas at 3 d, 6 retinas at 14 d, 4 retinas at 28 d, and 18 control retinas
10/C	GLUT3 increase in ON	n = 5 ONs at 6 h, 7 ONs at 3 d, 6 ONs at 14 d, 5 ONs at 28 d, and 19 control ONs
10/D	No change of GLUT3 in retina	n = 9 retinas at 6 h, 11 retinas at 3 d, 6 retinas at 14 d, 4 retinas at 28 d, and 18 control retinas

## Data Availability

The data is contained within the article and Supplementary Files.

## Supplementary Materials

The following supporting information can be downloaded at: www.mdpi.com/article/10.3390/antiox11050888/s1, Figure S1: Electrophoresis blots for GLUT1 and GLUT3.
