# Supplementary material for: Ocular Hypertension Results in Hypoxia within Glia and Neurons throughout the Visual Projection
Source: Antioxidants (Basel). 2022 Apr 29;11(5):888. doi: 10.3390/antiox11050888 (PMC9137916; doi:10.3390/antiox11050888)
Supplement: Supplementary file 1 [file antioxidants-11-00888-s001.zip › antioxidants-1675487-supplementary.pdf]

Supplementary Files for Protein Analysis

Protein analysis was undertaken using the Wes capillary electrophoresis instrument from Protein Simple. The output of the instrument can be shown as blot lanes in the manner of a western immunoblot. Shown below are the blot lane collections for Figure 9, the analysis of glucose transporter-1 and -3 in retina and optic nerve.

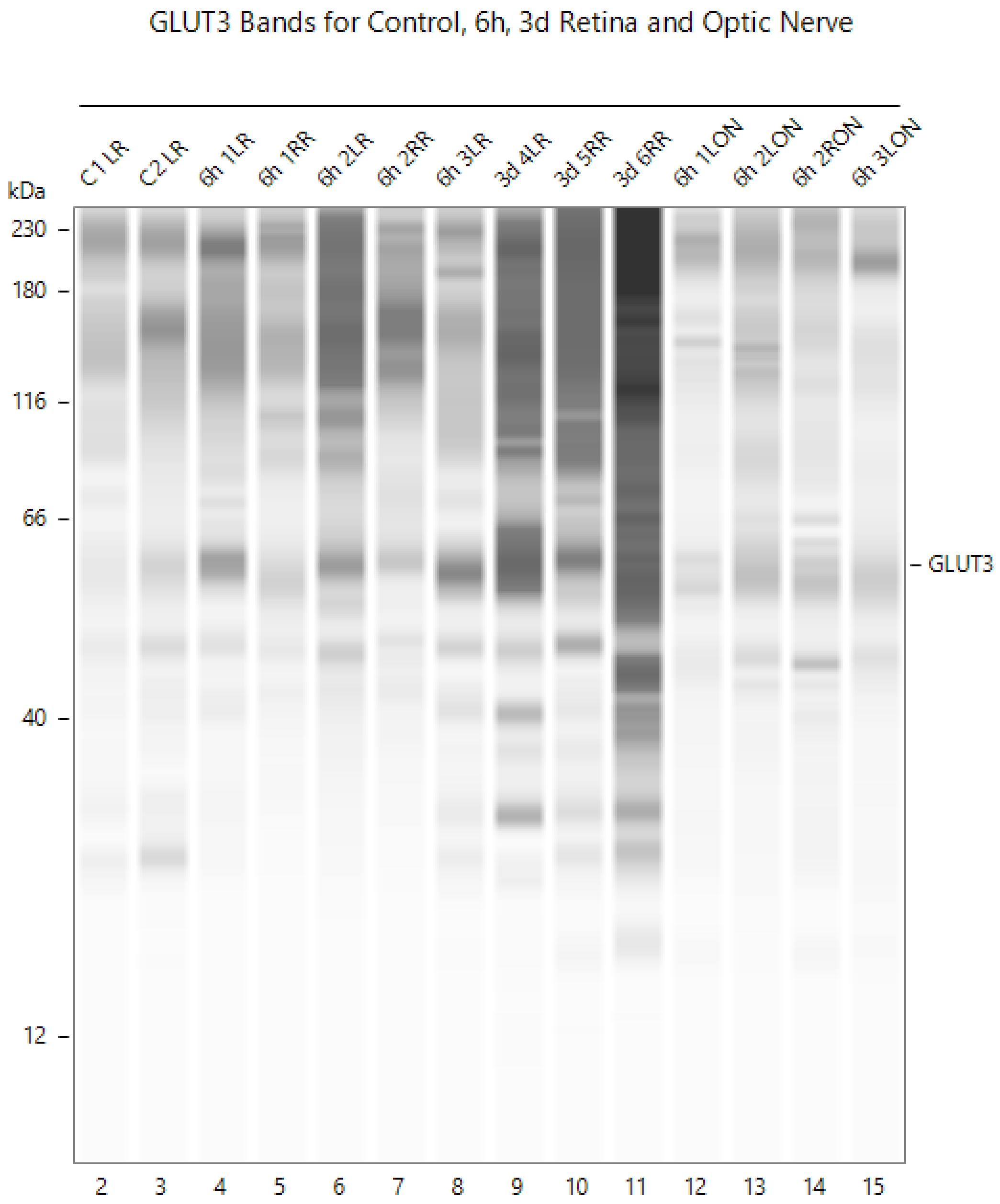

Control GLUT1 in Retina and Optic Nerve

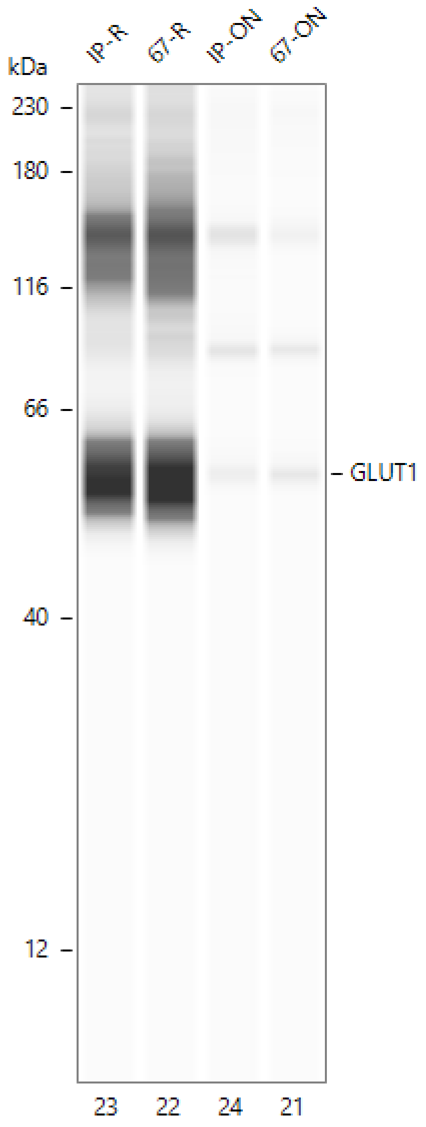

Figure S1: Electrophoresis blots for GLUT1 and GLUT3.
